# Grazing-incidence X-ray diffraction tomography for characterizing organic thin films

**DOI:** 10.1107/S1600576721007184

**Published:** 2021-09-04

**Authors:** Esther H. R. Tsai, Yu Xia, Masafumi Fukuto, Yueh-Lin Loo, Ruipeng Li

**Affiliations:** a Center for Functional Nanomaterials, Brookhaven National Laboratory, Upton, NY 11973, USA; b Department of Chemical and Biological Engineering, Princeton University, Princeton, NJ 08544, USA; c National Synchrotron Light Source II, Brookhaven National Laboratory, Upton, NY 11973, USA; d Andlinger Center for Energy and the Environment, Princeton University, Princeton, NJ 08544, USA

**Keywords:** grazing-incidence wide-angle X-ray scattering, GIWAXS, tomography, thin films, organic transistors

## Abstract

Characterization of thin films is of paramount importance for evaluating material processing outcomes/efficiency as well as establishing structure–property/performance relationships. This article introduces grazing-incidence diffraction tomography (GID tomography), a technique that combines grazing-incidence X-ray scattering and computed tomography to quantitatively determine the dimension and orientation of crystalline domains in thin films without restrictions on the beam coherence, substrate type or film thickness. This computational method extends the capability of synchrotron beamlines by utilizing standard X-ray scattering experiment setups.

## Abstract 2

A computational method that directly translates the scattering peak information to crystalline domain shapes and orientations is presented. The method is demonstrated at a synchrotron beamline with a standard X-ray scattering setup.

## 1. Introduction   

Characterization of thin films is essential for evaluating material processing outcomes and efficiency as well as establishing structure–property/performance relationships. X-ray scattering methods allow for the determination of morphological and molecular structures over a broad length scale, ranging from ångströms to micrometres or more. Grazing-incidence X-ray methods are often used to study thin-film properties for applications in, for example, semiconductors and photovoltaics (Hexemer & Müller-Buschbaum, 2015 ▸; Smilgies *et al.*, 2002 ▸; Zhang *et al.*, 2006 ▸; Jung *et al.*, 2019 ▸). Scattering data are often reduced and interpreted to determine statistically the phases or crystal orientations for bulk samples. It would be beneficial, however, if scattering data sets could provide access to the real-space spatial distribution of material characteristics. Kuhlmann *et al.* (2009 ▸) employed computed tomography (CT) coupled with grazing-incidence (GI) small-angle X-ray scattering (GISAXS) to topographically reconstruct self-assembled colloidal crystalline structures of polystyrene spheres and gold particles. Ogawa *et al.* (2015 ▸, 2020 ▸) reported the reconstruction of spatial maps of gold and platinum nanoparticles on silicon substrates through GISAXS coupled with CT. They also showed the reconstruction of organic–inorganic multilayers (Ogawa *et al.*, 2017 ▸) by utilizing the different total reflection angles and penetration depths between the organic and inorganic layers. These CT-based approaches achieved high-resolution reconstruction but required different structure factors to identify the various materials and were only applicable to in-plane isotropic films, *i.e.* powder. Freychet *et al.* (2019 ▸) utilized a rotational GISAXS method to determine the shape and the orientation of a line grating with sub-nanometre precision. The approach focused on the morphological structure instead of the spatial resolution. On the other hand, a coherent X-ray source allows for the direct reconstruction of domain spatial distribution from the scattering features without tomography. Sun *et al.* (2012 ▸) have demonstrated the reconstruction of nanostructure from coherent X-ray surface scattering. These grazing-incidence methods rely on significant heterogeneities in materials or structures in the film to reconstruct the corresponding spatial maps. It is also assumed that the in-plane scattering is isotropic and thus no information on molecular orientations is revealed. What is lacking is a tomographic method that can spatially resolve the orientations even for films with a homogeneous distribution of materials.

The organic transistor community has particular interest in investigating structures of thin films as these structures directly impact the device performance. Unveiling the hierarchical 3D structures of a macroscopic film remains a challenge – crystalline structure details, such as the molecular arrangement and molecular orientation, are often on the length scale of ångströms to nanometres, whereas domain size and orientational order can extend over scales from micrometres to millimetres. The interrelation between crystalline structure and morphology demands a characterization technique that covers a wide range of length scales. For example, the fast-evolving casting methods allow fine control of the structure and morphology of thin films, including the fine tuning of grain boundaries (R. Li *et al.*, 2012 ▸; Rivnay *et al.*, 2009 ▸), a variety of polymorphism (Giri *et al.*, 2014 ▸; Diao *et al.*, 2014 ▸), high in-plane anisotropy (H. Li *et al.*, 2012 ▸; Yuan *et al.*, 2014 ▸) and forming large single-crystalline domains (Minemawari *et al.*, 2011 ▸; Kim *et al.*, 2014 ▸; Makita *et al.*, 2020 ▸; Xia *et al.*, 2021 ▸). These thin films are often cast with homogeneous thickness of the same material with similar structure factors but varying in-plane orientations. What is needed is a method to characterize thin films with sufficient spatial resolutions both macroscopically for domain location and dimension and microscopically for crystal structure and orientation.

In this work we demonstrate the characterization of multiple domains on centimetre-sized organic semiconductor thin films using the grazing-incidence wide-angle X-ray scattering (GIWAXS) setup and data. Specifically, we introduce a computational analysis method, grazing-incidence diffraction tomography (GID tomography), to determine the shapes and absolute orientations of single-crystalline domains by leveraging knowledge of the reciprocal lattice. Projectional information is obtained through the elongated incident X-ray footprint on the sample and a tomographic data set is acquired by lateral scanning and rotational scanning. Differently from typical CT (Kak & Slaney, 2001 ▸) and reciprocal mapping for individual scattering units (Reiten *et al.*, 2015 ▸; Liebi *et al.*, 2015 ▸, 2018 ▸), our method utilizes the fact that peaks from a single crystal only appear on the detector when the reciprocal lattice intersects with the Ewald sphere and disappear when the crystal is rotated away from the intersection, offering information on the orientation of the crystal domain. Using only information on the indexed peaks and their corresponding rotation angles, domains are reconstructed to reveal the interrelation between the crystalline structure and morphology of thin films. This simple construct provides a direct and robust solution for spatially resolving crystalline domain shapes and orientations. The method takes advantage of the high photon flux and fast acquisition at synchrotron beamlines to obtain a large tomographic data set. Standard X-ray scattering setups without a coherent X-ray source can be used, allowing the method to be deployed easily and widely.

## 2. Method and material   

### 2.1. Grazing-incidence X-ray scattering   

**Figure 1:**
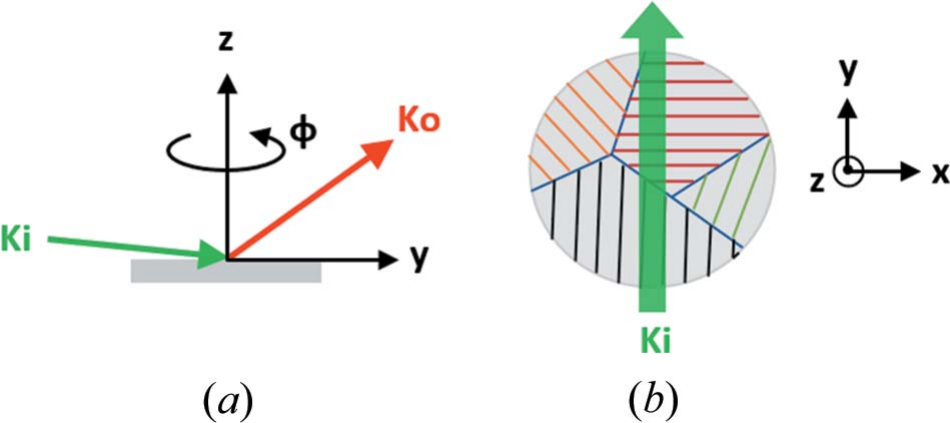
Experiment for characterizing multi-domain thin films. (*a*) GIWAXS measurement geometry. (*b*) An X-ray beam is scanned in the **x** direction at all azimuthal rotation angles ϕ to produce tomographic data. A given crystalline domain has a well defined in-plane orientation and thus the diffraction condition is satisfied only at a set of specific rotation angles.

Our goal is to determine the in-plane structure, including the multiple domain dimensions and orientation of each single-crystal domain, through GIWAXS measurements. A schematic of grazing-incidence experiments is shown in Fig. 1 ▸(*a*). The thin-film surface normal is along the *z* axis while the *yz* plane corresponds to the plane of incidence, with the *y* axis coincident with the beam propagation direction when the grazing-incidence angle is zero. The azimuthal rotation ϕ has is axis along **z**. For this work, GIWAXS data were collected at the 11-BM Complex Materials Scattering (CMS) beamline at the National Synchrotron Light Source II, Brook­haven National Laboratory. The samples were mounted on a stack of stages consisting of, from top to bottom, two in-plane translations, a pair of two tilt stages, a full rotation stage for ϕ, another pair of two tilt stages (pitch and roll), and the transverse and vertical translations. The upper double-tilt stages were used to orient the sample surface normal to the *z* axis, while the lower double-tilt stages were used to set the grazing-incidence angle and bring the *z* axis into the incidence plane. The upper in-plane translations are used to center the sample about the *z* axis, whereas the lower translations are used to adjust the sample height and translate the sample along **x**. The stages ensured the sample was aligned perpendicular to the rotation axis with an accuracy of 0.002°. Measurements were collected at 13.5 keV (λ = 0.9184 Å) with a 0.2 mm (horizontal) × 0.05 mm (vertical) beam and a grazing-incidence angle of ∼0.1°, giving a footprint of a few centimetres, larger than the typical sample dimensions. The collected scattering pattern therefore contains data from this elongated footprint and can be considered a ‘projection’ or a sum of sample characteristics along the X-ray path.

A GID tomographic data set includes scattering patterns collected with ϕ over 180 or 360° azimuthal rotation, similar to that in CT. However, single-crystal films have strong in-plane orientation and the scattering geometry for a certain GIWAXS peak is only met at a specific azimuthal rotation, as illustrated in Fig. 1 ▸(*b*). Each thin-film sample was scanned over *x* = −5 to 5 mm with a step size of 0.2 mm and with ϕ over 360° with a sample-stage rotation step of Δϕ = 0.5°. Each exposure time was 1 s, giving a total of 36 000 scattering patterns (∼130 GB) captured over around 12 h. A Pilatus 800k detector with a pixel size of 0.172 mm was placed 220 mm downstream of the sample to capture the GIWAXS data.

### 2.2. Characterization of organic semiconductors   

In this work, we examined two samples on different substrates to validate GID tomography. A sample on a transparent glass substrate (Sample G) allows us to compare the results between our method and transmission polarized light microscopy, as will be discussed in Section 2.3. GID tomography is also used to characterize a sample on a silicon substrate (Sample S) as Si is the standard substrate for organic transistor devices.

The organic transistor films were cast as a mixture of C8- and C12-BTBT ([1]benzothieno[3,2-*b*][1]benzothiophene) by a solvent-free coating method (Xia *et al.*, 2021 ▸). A mixture of C8-BTBT and C12-BTBT in the ratio of 1:1 was melted at 373 K to its liquid-crystalline phase and subsequently blade-cast to be a freely suspended and preferentially oriented membrane held in a metal cavity. This prealigned membrane was then transferred onto a glass substrate or polymethyl methacrylate-coated Si substrate and cooled at a rate of 0.1 K min^−1^ to a co-crystal phase of C8-C12-BTBT (BTBT). The crack-free films contained multiple large domains with sizes of up to several millimetres. This solvent-free coating method for creating large domains on thin films opens possibilities for large-scale fabrication of organic electronics.

**Figure 2:**
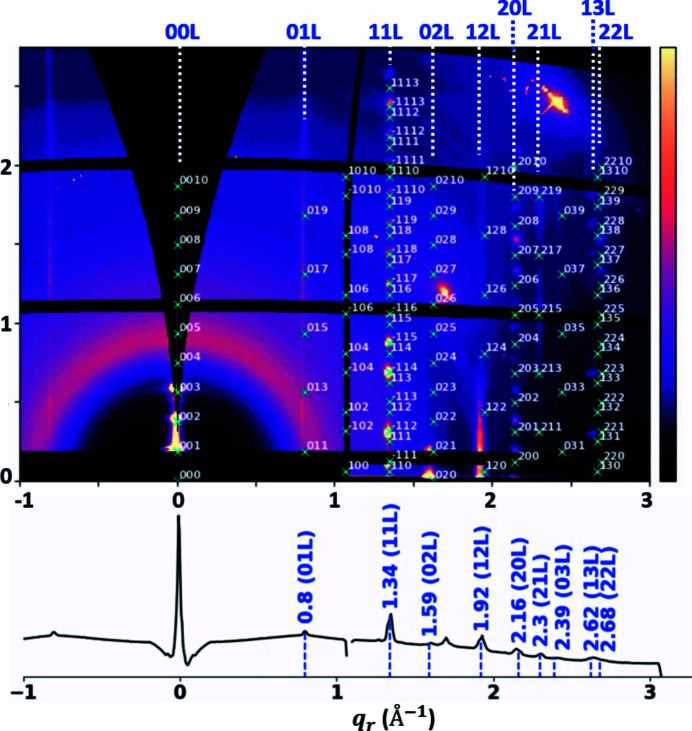
GIWAXS patterns summed over rotation angles with peaks indexed for BTBT. The crystal [001] direction is aligned along the *z* direction, *i.e.* it is normal to the substrate.

Fig. 2 ▸ gives the summed GIWAXS patterns for the Si-substrate sample from all rotation angles with peaks indexed, showing the preferred alignment of the *c* axis along the out-of-plane direction. The lattice parameters determined by this indexing are *a* = 5.8, *b* = 7.72, *c* = 33.7 Å, α = 90, β = 93.2, γ = 90°, space group *P*21/*c*. It is assumed that there are no polymorphs. The results indicate that all domains share the same out-of-plane orientation but they vary in the in-plane orientation. The size of these large domains cannot be estimated by the Scherrer equation as the beam size is smaller than the domain size. For instance, for a 1 mm domain and with shape factor *K* = 0.9, at *q* = 0.8 Å^−1^ (*i.e.* Bragg angle θ_B_ = 3.35°) the line broadening would be 5 × 10^−6^°. This is much smaller than the instrument limit of 0.067°, which originated from pixel broadening (0.04°), beam divergence (0.006°) and beam size (0.05°). The broadening of the in-plane peaks instead can be attributed to the elongated X-ray footprint on the sample.

### 2.3. Polarized optical microscopy   

Molecular orientations can be determined through measuring birefringence, which is the anisotropy of refraction. Light polarized in different directions travels at varying speeds along the fast and slow axes of a molecule, resulting in a differential phase shift called retardance. The slow axis is the principal axis with the largest refractive index. Birefringence imaging through polarized optical microscopes (Oldenbourg & Mei, 1995 ▸; Oldenbourg, 1996 ▸) allows for the determination of the retardance and the orientation of the slow axis. A raw data set includes grayscale intensity images acquired with polarized light of different axis orientations, achieved by varying the retarder settings. This set of images is then used by a post-processing algorithm to compute the retardance magnitude and orientation (Mehta *et al.*, 2013 ▸). Careful calibrations and background correction are necessary for the accurate determination of the retardance and orientation. The results are high-resolution (sub-µm) images of these material properties. In this work, the polarization microscopy was carried out on a Zeiss Axio Imager M2m microscope at a wavelength of 546 nm. Multiple fields-of-view (FOVs) were acquired and stitched together to examine the entire film.

### 2.4. GID tomography   

**Figure 3:**
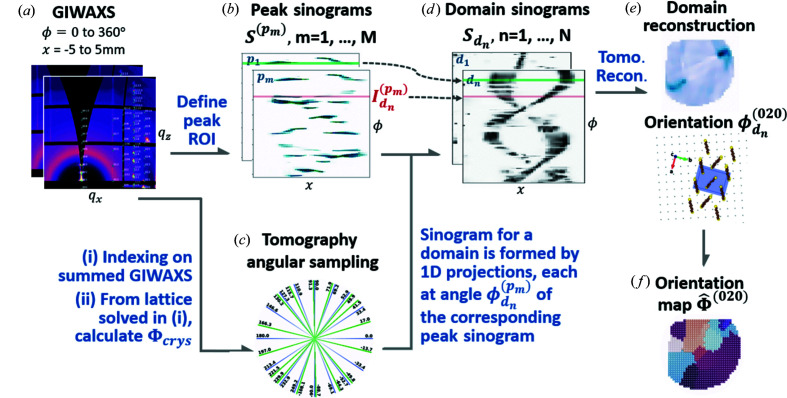
Overview of GID tomography. (*a*) GIWAXS patterns are collected while scanning in *x* and rotation angle ϕ and subsequently used to form (*b*) peak sinograms for each peak, *e.g.* (011), (020). (*c*) On the basis of the indexing of GIWAXS patterns, a series of azimuthal rotation angles are calculated, each corresponding to a peak. (*d*) The sinogram for domain *d*_*n*_ is then formed from 1D projections 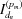, each at angle 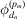 of the corresponding peak sinogram for peak *p*_*m*_. From the domain sinograms in (*d*), tomographic reconstruction methods can then be used to recover (*e*) the shape of each domain on (*f*) the entire thin film.

An overview of GID tomography is given in Fig. 3 ▸. Tomographic GIWAXS data are collected along the **x** direction and at rotation angles ϕ, as shown in Fig. 3 ▸(*a*), and subsequently used to form peak sinograms for each peak, shown in Fig. 3 ▸(*b*). The tomographic angles at which projections of a domain show up, illustrated in Fig. 3 ▸(*c*), are calculated from the lattice parameters, which are obtained by indexing the summed GIWAXS data. As shown in Fig. 3 ▸(*d*), a sinogram for domain *d*_*n*_ is then formed from a series of 1D projections, each at angle 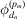 of the corresponding peak sinogram for peak *p*_*m*_, where *d*_*n*_ refers to one of the *N* domains in the sample and *p*_*m*_ refers to one of the *M hkl* peaks that can be observed from the 2D WAXS data. From the domain sinograms, tomographic methods can be used to reconstruct the shape and orientation of each domain, as shown in Figs. 3 ▸(*e*) and 3 ▸(*f*).

Differently from typical CT data, projections of the domain are not observed at all rotation angles. As the sample rotates, information about the domain appears on the scattering pattern when the Bragg condition is satisfied with the corresponding in-plane and out-of-plane angles. An example of the raw GIWAXS data is given in the supplementary video, in which peaks can be seen appearing and disappearing as the sample rotates. The intensities of different peaks depend on, for example, the molecular form factor and structure factor, the instrumental resolution, and the proximity to the intersection of the reciprocal lattice and Ewald sphere. The peak intensity is also correlated with the domain size, film thickness and crystallinity. Instead of performing reciprocal mapping for an individual scattering unit (Reiten *et al.*, 2015 ▸; Liebi *et al.*, 2018 ▸, 2015 ▸), our method translates the peak information from scattering data to domain-orientation maps. In this work we do not need to consider peak intensity variations due to the aforementioned factors but only consider the appearance of a peak, which indicates the detection of a domain in the corresponding orientation.

Assuming the thin-film thickness is homogeneous, the overall film shape is denoted as *O*(*x*, *y*), which is a binary mask indicating the existence and spatial extent of the thin film. At each pixel (*x*, *y*), the existence of the film is indicated by 1; otherwise the pixel is assigned value 0. Similarly, the spatial distribution of each crystal domain is given by the binary mask 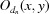. Each domain can be represented by its orientation. For notational simplicity, domains with the same orientation are denoted as the same domain, *d*_*n*_. This does not imply that domain *d*_*n*_ is a single domain but it can represent domains at multiple locations with the same orientation. For domain *d*_*n*_, in-plane peak *p*_*m*_ appears on the scattering pattern only when the sample is rotated to the corresponding angle 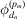. The 1D projection of crystal domain *d*_*n*_ at rotation angle 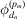 is the intensity at the region-of-interest (ROI) of peak (*p*_*m*_) on the scattering pattern, given by 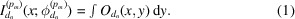
This 1D projection has dimensions of 1 by *N*_*x*_, where *N*_*x*_ is the number of positions measured along *x*. The ROI intensities for peak *p*_*m*_ at all *x* and ϕ provide information on all the domains, illustrated by the peak sinogram: 
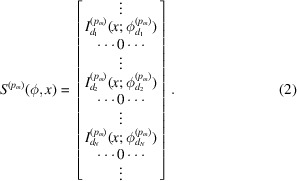
The peak sinogram has dimensions of *N*_ϕ_ by *N*_*x*_, where *N*_ϕ_ is the number of rotation angles used in the experiment and depends on Δϕ. The maximum number of domains that can be identified is *N* = *N*_ϕ_ for ϕ = [0, 180] and *N* = *N*_ϕ_/2 for full rotation ϕ = [0, 360].

Since all the domains have the same out-of-plane orientation, the peak sinogram for out-of-plane peaks, *e.g.* (002), allows for the reconstruction of the overall thin-film shape, 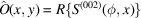, where *R* is a standard tomographic reconstruction method, such as filtered back projection (Kak & Slaney, 2001 ▸) or regridding methods (Dowd *et al.*, 1999 ▸; Marone & Stampanoni, 2012 ▸). A tomographic reconstruction takes object projections collected at different rotation angles and assembles them in either the real space or reciprocal space to reconstruct the object.

For in-plane peaks, if we can attribute each row component of 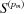 to the corresponding domain, as shown in equation (2), and assemble a domain sinogram 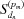 from each peak for the same domain, we can reconstruct the spatial map of the domain and, with the angles 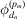, also determine quantitatively its crystal orientation. The domain sinogram can be formed by collecting the 1D projection from each *hkl* peak, 
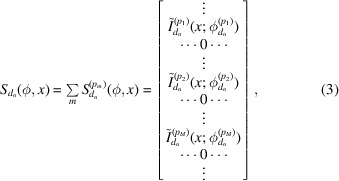
where 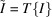 and *T* is the transformation applied to the array. For *T* in this work, we simply use normalization with respect to the maximum of the row because the absolute peak intensity or the intensity variation between peaks is not used in the method; only information on the appearance of peaks due to the detection of the corresponding domain orientation is needed. Section 2.5 describes in detail the data processing procedures.

Many rows of the sinogram in equation (3) are zero because there are no peaks at rotation angles that satisfy the Bragg condition. The key to the formation of the domain sinogram is knowing the 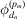 for each *p*_*m*_ peak. The azimuthal angles between (*hkl*) planes can be calculated as shown in Section 2.6. With 020 peak orientation as the reference angle, the list of angles at which a domain sinogram has nonzero elements can be expressed as 
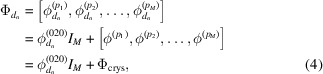
where *I*_*M*_ is an *M*-dimensional array filled with ones and 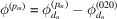. The crystal rotation list, Φ_crys_, can be calculated from the crystal lattice parameters and the X-ray wavelength. As a result, the orientation of the domain can be represented by a scalar, 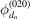. Once the crystal rotation list Φ_crys_ and domain sinogram in equation (3) are determined, a standard tomographic reconstruction method of choice (Kak & Slaney, 2001 ▸) can be used to reconstruct each domain.

To visualize the domain shapes and orientations of a thin film, we assume that there is only one domain at each pixel (*x*, *y*) and represent each domain by its orientation angle 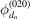. All reconstructed domains must be considered to determine the orientation angle at a pixel. Here we apply normalization to each 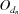 and, for each pixel, choose the domain *d*_*n*_ that gives the largest reconstructed value to be the domain at the pixel. The reconstructed ‘orientation map’ for the thin film is then given by 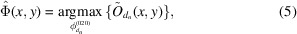
where 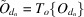 is the domain reconstruction after a transformation *T*_*o*_, *e.g.* normalization used in this work or segmentation methods. Thin films with overlapping domains can be expressed in terms of two orientation maps by choosing a suitable *T*_*o*_ and selecting the two domains that give the largest two 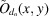 at each pixel.

### 2.5. Data calibration   

The intensities from each scattering pattern and especially for each peak are different and thus it is natural that calibration or normalization should be considered when forming the domain sinogram in equation (3). In GID tomography, we only focus on whether the peak showed up or not, which directly implies the specific crystal orientation that met the Bragg condition. Thus, the calibration for removing background noise or intensity normalization does not have to be as rigorous as typical scattering data analysis where interpretations are based on the scattering intensity. For removing background and noise, we simply define an ROI with no obvious peaks and use it for background subtraction; for intensity calibration, we apply normalization with respect to the maximum along the *x* scan. We find that these simple procedures are sufficient to generate meaningful sinograms for each domain. This shows that the method is robust to data noise of almost any kind – as long as we can identify the peaks on the scattering pattern.

On the other hand, the background, mainly from the substrates, needs to be separated from the scattering signal. An amorphous glass substrate gives a broad ring on the scattering pattern in Fig. 6(*a*1) below[Sec sec3]. Even though this ring overlaps with the BTBT peaks, the background intensity at a grazing-incidence angle below the substrate critical angle is negligible compared with the peak intensities and thus the aforementioned background removal method was effective. The silicon peaks in Fig. 6(*a*2) are sharp and isolated and do not shadow or overlap with the crystal peaks that we are interested in. The peaks allow the determination of the thin-film orientation relative to the Si wafer. In short, as long aswe can identify the peaks from the scattering pattern, the background or noise does not interfere with the orientation-angle reconstructions.

### 2.6. Tomographic angular sampling   

In typical CT, a projection of the sample is captured on the detector at every step of the sample-stage rotation. This rotation step size, Δϕ, determines the reconstructed image resolution for CT. However, for in-plane GID tomographic data, domain projections are only obtained at specific angles, as shown in equation (4). The tomographic angular sampling is thus determined by Φ_crys_ instead of the rotation step Δϕ. While the step size Δϕ determines the sensitivity of detecting a peak on the detector and of detecting small variations in domain orientation angles, the tomographic angular sampling Φ_crys_ determines the transverse in-plane spatial resolution for the shapes of reconstructed domains.

**Figure 4:**
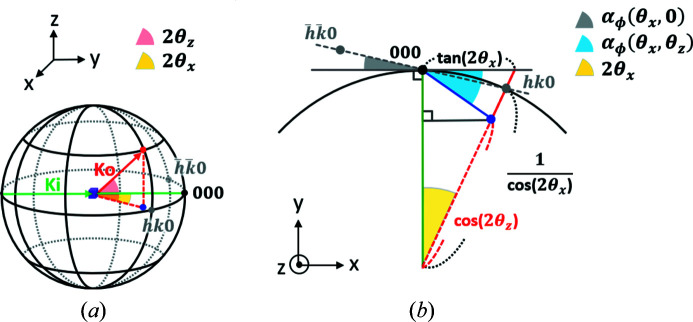
Illustration of azimuthal rotation in (*a*) three dimensions and (*b*) top view. The scattering occurs when the reciprocal lattice intersects with the Ewald sphere (radius = 1). The crystal rotation needed for the intersection is given for 2D (gray dot and angle) and 3D cases (blue dot and angle). The blue dot is the projection of the (*hkl*) lattice onto the Ewald sphere on the *xy* plane. With given scattering angles θ_*x*_ and θ_*z*_, the azimuthal rotation α_ϕ_ between planes can be calculated.

The crystal rotation list Φ_crys_ is determined by calculating the azimuthal rotation angles between crystal planes (Prince, 2004 ▸; Breiby *et al.*, 2008 ▸). Fig. 4 ▸ gives an illustration of the scattering condition, *i.e.* when the reciprocal lattice intersects with the Ewald sphere with azimuthal rotation. The azimuthal rotation angle between (*hkl*) and 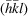 can be calculated from the scattering angles in the azimuthal and *q*_*z*_ directions. As shown in Fig. 4 ▸(*b*), the reciprocal lattices (*hk*0) and 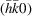 intersect with the Ewald sphere when rotated azimuthally by α_ϕ_ and −α_ϕ_, respectively. Therefore, in 2D cases, the azimuthal rotation between (*hk*0) and 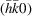 is simply 2α_ϕ_ = 2θ_*x*_. In 3D cases, *i.e. q*_*z*_ ≠ 0, the lattice needs to rotate an additional distance, as illustrated by moving from the gray dot to the blue dot in Fig. 4 ▸. The azimuthal rotation can be calculated from the scattering angles θ_*x*_ and θ_*z*_ as
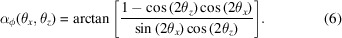
The azimuthal angle between peaks in the same series, *e.g.* 11*l* with *l* = 1, 2,…, is then 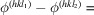 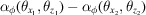. The incident angles are small for the grazing-incidence experiments considered here and are thus negligible for calculations. Cases with non-negligible incident angles are discussed by Breiby *et al.* (2008 ▸). For illustration purposes, (*hk*0) is plotted on the *xy* plane in Fig. 4 ▸, whereas it is actually slightly off the plane because the crystal is monoclinic.

**Figure 5:**
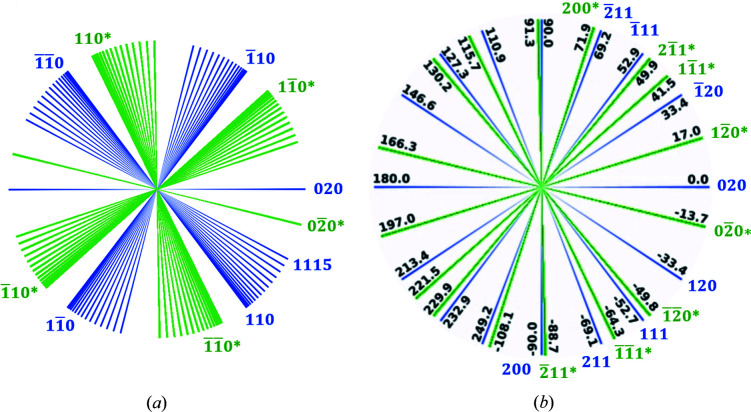
Crystal rotation list for BTBT Φ_BTBT_, which consists of azimuthal rotation angles between peaks. Each peak contributes to a tomographic projection. The quality of a domain reconstruction depends on this tomographic angular sampling. The blue lines and text show the peaks with positive *q*_*x*_ in the scattering pattern, and green and suffix ‘*’ are used for peaks with negative *q*_*x*_. The angles for the peak series 11*L* in (*a*) show the broad angle range that can be covered by a series. Selected peaks in (*b*) show that there is no large missing wedge for tomography.

Fig. 5 ▸ shows the crystal rotation list for BTBT Φ_BTBT_, which is calculated from the BTBT lattice parameters and equation (6). The angle at which (020) scattering occurs is used as reference, *i.e.* ϕ^(020)^ = 0, following the definition in equation (4). The blue lines and text are for peaks that appear at positive *q*_*x*_, while green and the suffix ‘*’ are used for peaks with negative *q*_*x*_. For example, peak *hk*0 in Fig. 4 ▸(*b*) is projected to positive *q*_*x*_ on the scattering pattern and 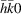 towards negative *q*_*x*_ after a counterclockwise 2α_ϕ_ rotation. When the crystal rotates by 180°, the 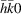 peak is then observed at positive *q*_*x*_. As shown in Fig. 5 ▸, it is important to use data from as many peaks as possible for better tomographic angular sampling, as each peak corresponds to a projection 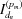, which contributes to the nonzero components in the domain sinogram 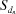. Peaks in the same series can also contribute to a broad range of angles, *e.g.* the rotation angle between 110 and 1 1 15 is 23°, as illustrated in Fig. 5 ▸(*a*). Fig. 5 ▸ highlights the significance of using all identifiable peaks in the scattering pattern for improved tomographic angular sampling.

For an object with diameter *D*, the required number of tomographic projections to achieve isotropic resolution Δ*x* is given by 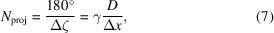
where Δζ is the corresponding angular step for uniform angular sampling and γ is around 1.57 according to Crowther’s criterion (Crowther *et al.*, 1970 ▸; Kak & Slaney, 2001 ▸). In conventional tomography, the tomographic angular step Δζ equals the rotation step Δϕ. For GID tomographic data of single crystals, the tomographic angular sampling is determined by the crystal rotation list Φ_crys_, with each angle in Φ_crys_ corresponding to the appearance of an *hkl* peak. As a result, the tomographic angular sampling depends on the number of peaks that can be covered by the 2D scattering patterns, *i.e.* the *q* range in both *q*_*x*_ and *q*_*z*_. It is, therefore, advantageous to place the detector at one or multiple locations where most peaks can be captured. It is also ideal for Φ_crys_ to provide an angular sampling that is almost uniform or at least without missing data over a large range of angles. A transverse resolution of Δ*x* on the *xy* plane can be achieved if Φ_crys_ offers similar angular sampling to Δζ.

## 3. Results   

In this work we present results for two samples to demonstrate the direct reconstructions of domain shape and orientation in the grazing-incidence geometry. Sample G has an optically transparent glass substrate, which allows for validation of domain orientations through polarized optical microscopy, while the results for Sample S show that our method allows for domain reconstructions for a nontransparent silicon substrate.

**Figure 6:**
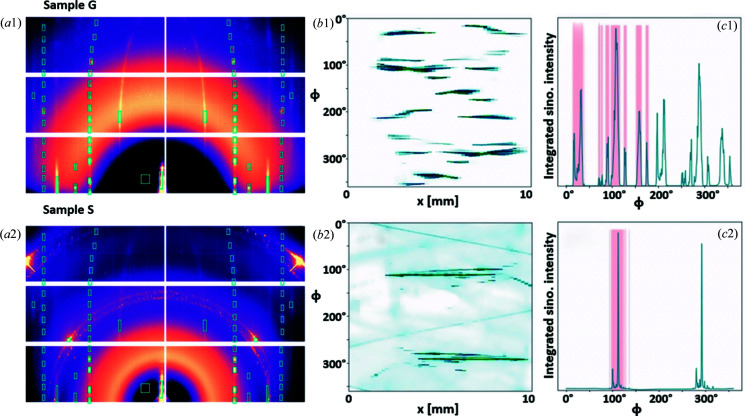
Scattering data and peak sinograms. (*a*) Summed GIWAXS patterns with peak ROIs for the glass-substrate sample (top row) and the silicon-substrate sample (bottom row). (*b*) Peak sinograms for the 020 peak and (*c*) their integrated 1D intensity curves. Pink highlights indicate the existence of domains.

In the co-crystal of C8- and C12-BTBT the two molecules are arranged on the same lattice and form an expanded unit cell from a single component. The lattice parameters are calculated from the indexed peaks (Smilgies & Blasini, 2007 ▸) on the summed GIWAXS pattern in Fig. 2 ▸. For each peak, an ROI is defined on the summed GIWAXS pattern to ensure that the ROI is large enough to capture peaks contributed by the upstream edge of the film as well as the downstream edge of the film. Fig. 6 ▸(*a*) shows the summed scattering pattern with peak ROIs indicated in cyan boxes. For each peak, the intensities inside the ROI are summed and subsequently used to form the peak sinogram 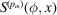. The 020 peak sinograms are shown in Fig. 6 ▸(*b*) and the 1D integrations of the sinograms in Fig. 6 ▸(*c*). The 1D curve allows us to identify the number of domains in the thin film.

### 3.1. Sample-shape reconstruction   

**Figure 7:**
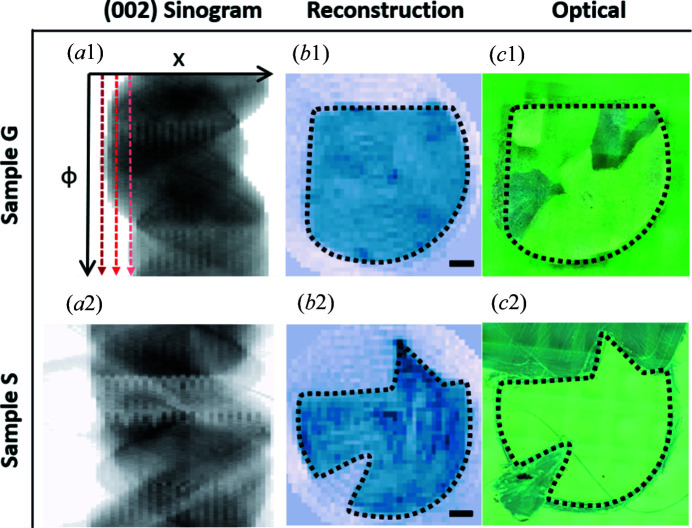
Reconstruction of sample shapes. (*a*) The 002 peak sinogram and (*b*) its reconstruction, compared with (*c*) the optical micrograph for Sample G (top row) and Sample S (bottom row). For each sample, the same dashed contours are plotted on the images, which shows that with the out-of-plane data we can reconstruct the sample shape and determine the center of rotation. The dark to light red lines in (*a*1) show the scanning direction. No significant intensity changes along the scanning direction were observed, indicating negligible radiation damage. Scale bars are 1 mm.

From the GIWAXS patterns in Fig. 6 ▸(*a*), we see that BTBT is always oriented with (00*L*) normal to the substrate. Therefore the sinogram formed by the 00*L* peaks or a subset of the 00*L* peaks can be processed as conventional CT data to determine the sample shape. Figs. 7 ▸(*a*) and 7 ▸(*b*) show the sinogram and corresponding reconstructions using the 002 sinogram through a regridding method, Gridrec (O’Sullivan, 1985 ▸; Dowd *et al.*, 1999 ▸). In this tomographic reconstruction method, the Fourier transform of each 1D projection is interpolated onto the Cartesian grid and stitched together with data from all angles in the 2D Fourier space. An inverse 2D Fourier transform is then performed to recover the 2D object. In this work, we use the Gridrec implementation in the Python toolbox *TomoPy* (Gürsoy *et al.*, 2014 ▸) for reconstructions. Optical micrographs obtained from Keyence VHX-S650E with crossed polarizers are shown in Fig. 7 ▸(*c*) for comparison. Dashed contours for each sample are plotted in Figs. 7 ▸(*b*) and 7 ▸(*c*) to aid the comparison, showing successful reconstruction of the sample shapes.

The lateral resolution of sample-shape reconstructions is mainly limited by the beam size and the scanning step in the **x** direction. It is typical for tomography experiments to aim for an isotropic resolution. Therefore, with a beam size of Δ*x* = 0.2 mm and a sample size of *D* = 10 mm, it is ideal to have 71 projections over 180° according to equation (7), which corresponds to an even tomographic angular sampling Δζ = 2.3°. For reconstructing sample shapes with 002 peak sinograms, the tomographic angular sampling is simply Δϕ = 0.5°, which is much finer than the ideal sampling, 2.3°, and thus the reconstructed image resolution was not limited by the angular sampling. The reconstructed intensity should correspond to the crystallinity and film thickness, but it is also susceptible to background noise in the scattering pattern. Here we are only interested in determining the center of rotation and forming a mask with the sample shape for subsequent reconstructions of domain shapes and orientation angles.

In addition, the 002 sinograms can be used as an indication for observing radiation damage of the samples or the lack thereof. The dark to light red lines in Fig. 7 ▸(*a*1) illustrate the scanning direction from left to right (*i.e.* increasing *x*). No significant changes in the 002 peak intensity as the sample was scanned were observed. Thus the radiation damage is considered negligible for our imaging purposes. The solvent-free deposition method may have minimized the radiation damage as no residual solvent was trapped in the film.

### 3.2. Domain-shape and orientation reconstruction   

To reconstruct the shape and orientation of domains, first an ROI is defined for each observable *hkl* peak on the GIWAXS pattern to form a peak sinogram, given by equation (2). Subsequently, as shown by equation (3), domain sinograms are constructed by rearranging the rows of peak sinograms according to the crystal rotation list Φ_crys_. Interestingly, it is not always necessary to separate all peaks on the scattering pattern when defining peak ROIs. Peaks from the 11*L* series and 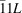 series are in close proximity to each other, as shown in Fig. 2 ▸. However, since the azimuthal rotation angle at which a peak appears differs for all peaks, we can use the same ROI for 110 and 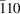, for instance. Figs. 5 ▸(*a*) and 8 ▸ show the large angle difference, around 106°, between the two series. Conversely, for peaks 110 and 111, different ROIs need to be defined since the rotation angles for these two peaks are close, as shown in Fig. 5 ▸(*a*). In short, depending on the rotation angle difference between the peaks, the peak ROI may or may not need to be defined precisely and the *q*-space resolution requirement might not be so stringent.

**Figure 8:**
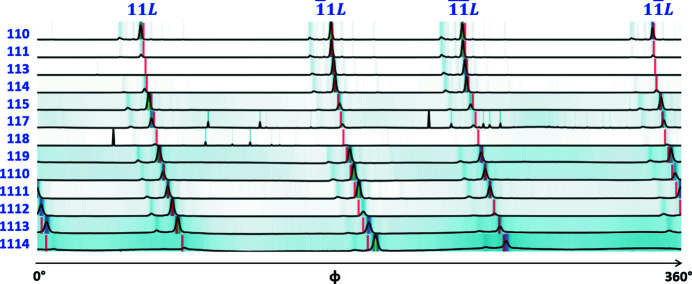
Integrated peak sinograms for Sample S: the same as that shown in Fig. 6 ▸(*c*) but for the 11*L* family. Red bars are theoretical angles calculated from the lattice parameters for BTBT, shown in Fig. 5 ▸.

To verify the calculated Φ_BTBT_, the peak sinograms are closely examined. As shown in Figs. 6 ▸(*b*2) and 6 ▸(*c*2), there is one large domain for Sample S. We can determine the crystal rotation list experimentally by tracking the angles at which peaks show up for this domain. Fig. 8 ▸ gives the 1D integrated sinograms for the 11*L* series for this sample. The angle difference between peaks corresponds to the difference between components in the crystal rotation list. The theoretical Φ_BTBT_ in Fig. 5 ▸ are shown as red lines in Fig. 8 ▸, matching well with the experimental data.

The maximum number of domains that can be characterized depends on the rotation step size Δϕ, since we define a domain as regions with the same orientation. As we can see from the 020 peak sinogram in Fig. 6 ▸(*b*), there are many near-zero components. By integrating the peak sinogram along **x**, we obtain a 1D curve of intensity versus ϕ, as shown in Fig. 6 ▸(*c*). The 1D curve allows us to identify the rotation angles at which the peak is observed, indicating the existence of a domain at the rotation angle. This is shown by the pink shading in Fig. 6 ▸(*c*), where angles between 0 and 180° with intensities greater than 1% of the maximum intensity are highlighted and considered for reconstruction. Thus, the number of angles under these highlights equals the number of reconstructed domains. In other words, in order to achieve a robust solution for equation (5), we reduce the number of possible domains to 153 and 58 for Sample G and Sample S, respectively. Many of these domains are slightly rotated compared with each other, and thus visually the number of domains in the reconstruction will appear to be much lower.

**Figure 9:**
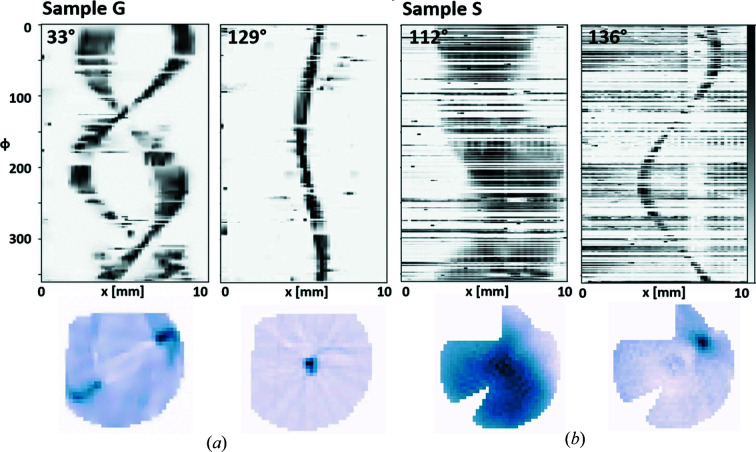
Examples of domain sinograms for (*a*) Sample G and (*b*) Sample S and their corresponding tomographic reconstructions. The angle labels give the orientation angle 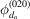 for the domain, as defined in equation (4).

**Figure 10:**
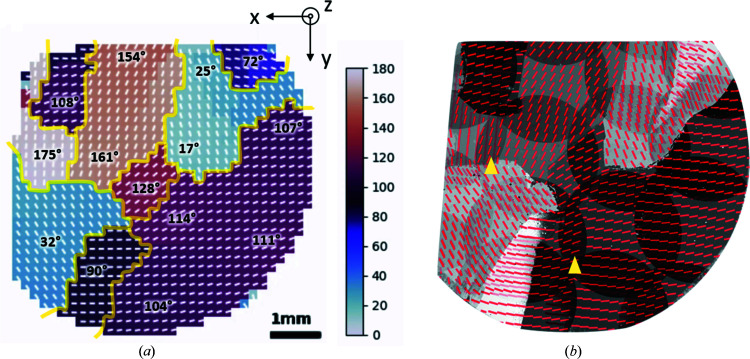
(*a*) Reconstructed domain orientation map, as defined in equation (5), for Sample G. Boundaries for major domains are highlighted in yellow. (*b*) Comparison with orientation map determined by polarized optical microscopy, showing high similarity. The yellow arrows in (*b*) show examples of where the orientation is different between the stitched images.

**Figure 11:**
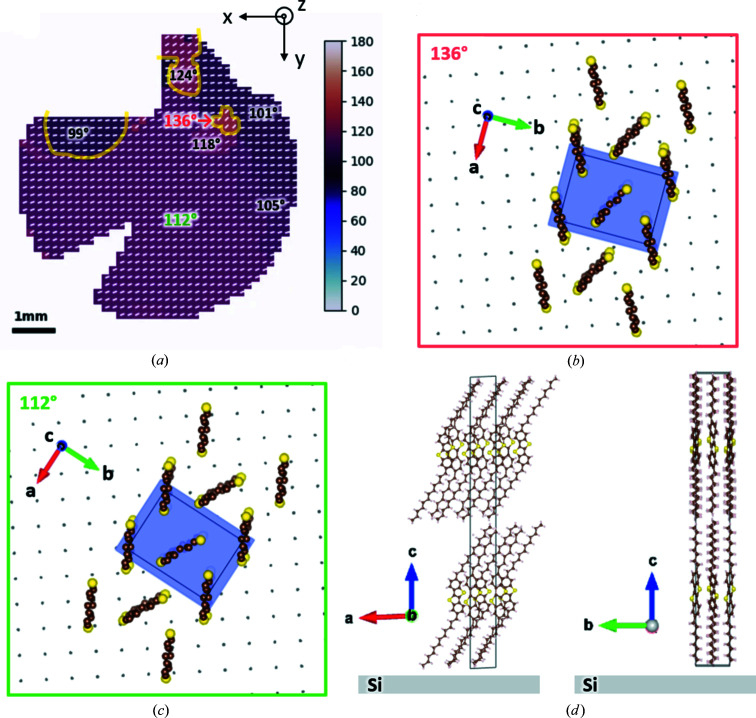
(*a*) Reconstructed domain orientation for Sample S. (*b*), (*c*) Illustrations of crystal orientations relative to the silicon substrate for two regions in (*a*). The gray dots show the atomic lattice of the (001) plane for silicon. (*d*) Side view of BTBT, where the *c* axis is aligned with the *z* axis.

Domain sinograms were constructed from the peak sinograms using the angles from the crystal rotation list. Each domain sinogram has nonzero components at 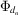 and linear interpolation was applied to fill up the data at missing angles. The number of projections here was *M* = 72 over 180°. Even with a limited number of projections, the reconstructions show the spatial distribution of each domain. Fig. 9 ▸ shows examples of domain sinograms and their corresponding reconstructions. The masks from Fig. 7 ▸ were used. The domain reconstructions for Sample S are successful despite the high background noise. Figs. 10 ▸ and 11 ▸ show quantitative reconstructions of the orientation maps for Sample G and Sample S, respectively, represented by the orientation of the 020 peak. It is assumed that each pixel can only have one crystal domain, and equation (5) was applied to determine the orientation of 020 at each pixel. When the boundaries of major domains are defined as having an orientation between neighboring pixels that varies by more than 10°, there are nine major domains for Sample G in Fig. 10 ▸(*a*). Major domain boundaries are highlighted in yellow. Within each of these major domains, the orientation angles vary gradually, implying distortion or lattice curvature. In Fig. 11 ▸(*a*), there was one large domain for Sample S, with smaller domains towards the upper edge.

To compare and validate our reconstruction results, we examined Sample G with polarized optical microscopy, as shown in Fig. 10 ▸(*b*). The orientations at several overlapping FOVs are different, highlighted by the yellow arrows. This discrepancy might be due to the imperfect microscope focusing or background correction. Since the orientation is computed from several micrographs, the accuracy of the orientation relies on micrograph intensities being carefully calibrated. Nonetheless, polarized microscopy allows for fast imaging of domain orientations in high resolution. The high degree of similarity between Figs. 10 ▸(*a*) and 10 ▸(*b*) demonstrates the validity of GID tomography. Unlike polarized microscopy methods, GID tomography directly uses GIWAXS data which contain crystal structure and orientation information. The method can also be applied to samples on a nontransparent substrate, such as our second sample with silicon. We can determine the orientation of the thin film relative to the silicon substrate, as well as the orientation of the substrate relative to the beam direction, as shown in Fig. 11 ▸(*d*). The molecular orientations for Sample S are illustrated in Figs. 11 ▸(*b*)–11 ▸(*d*), where the yellow spheres are sulfur atoms, brown denotes carbon atoms or carbon–sulfur bonds, and pink denotes hydrogen and carbon–hydrogen bonds. Not all atoms and bonds are shown for figure simplicity. The π–π stacking directions for charge transportation can also be identified through in-plane molecular orientations.

## 4. Discussion   

### 4.1. Resolutions and limitations   

The quality of domain reconstructions can be evaluated by the transverse 2D spatial resolution of domain shapes and the sensitivity of orientation angles between domains. The 2D spatial resolution of reconstructed domains depends on both the lateral resolution and the tomographic angular sampling. Here the lateral resolution Δ*x* equals the X-ray beam size for a non-coherent beam. With Δ*x* = 0.2 mm and *D* = 10 mm, the angular sampling required to achieve an isotropic resolution of Δ*x* is 71 projections over 180°, *i.e.* Δζ = 2.3°. For typical tomographic data sets that capture object projections at every rotation angle, the tomographic angular sampling equals the rotation step size. In this work, Δϕ = 0.5°, which is finer than the required Δζ. For GID tomography which utilizes the scattering peak information of single crystals for reconstruction, the angular sampling depends on the crystal rotation list Φ_crys_, as discussed in Section 2.6. For the scattering data acquired in this work, as shown in Fig. 6 ▸, multiple peaks were identified to provide 72 projections for the tomographic reconstruction, which is comparable to the aforementioned 71 projections in typical tomography. Although the angles between projections were not all equal for GID tomography, the plots of Φ_crys_ in Fig. 5 ▸ show that the full rotation was covered and that the nonuniform angular sampling did not result in missing data over a broad range of angles.

To enhance the 2D spatial resolution of domain reconstructions, both the lateral resolution and the tomographic angular sampling need to be improved. The lateral resolution can be adjusted by using a smaller beam and a smaller scanning step in *x*. The angular sampling depends on the number of observable peaks on the scattering pattern and thus it can be improved by changing the detector position, detector–sample distance or X-ray wavelength to allow more peaks and/or a broader *q* space to be captured on the detector. With knowledge about the crystal structure, we can also calculate the theoretical Φ_crys_ to predict which peaks are needed to improve the angular sampling and place the detector accordingly at the optimal location or scan multiple detector positions. On the other hand, the selection of *q* range during data acquisition is also critical to obtain a crystal rotation list that covers a broad rotation range and offers an angular sampling that is close to uniform sampling. For this work, the *q* range was selected to be *q*_r_ = [−2, 2] Å^−1^, as shown in Fig. 6 ▸. This scattering pattern was nearly symmetric in the sense that the beam center (*q*_r_ = 0) was close to the center of the scattering pattern. This was not the case for Fig. 2 ▸, where the beam center was offset towards negative *q*_r_ and thus covered *q*_r_ = [−1, 3] Å^−1^. The symmetric scattering pattern was chosen on purpose as data at higher scattering angle are more prone to lower signal-to-noise ratio, instrumentation resolution effects and elongated scattering due to the X-ray footprint on the sample. The symmetry of the pattern therefore ensures better quality data, and it in fact does not provide redundant information but provides projections at different rotation angles, as shown by the blue and green sets in Fig. 5 ▸.

Even though the rotation step size Δϕ does not directly affect the tomographic angular sampling, it still needs to be small enough to detect the crystal peaks as the reciprocal lattice rotates and intersects with the Ewald sphere. The rotation step size here was ΔΦ = 0.5°, which was much smaller than the typical orientational distribution in single crystals (Minemawari *et al.*, 2011 ▸; Schweicher *et al.*, 2021 ▸). In this work with the BTBT films, each peak spans about 1°, and thus the step size Δϕ = 0.5° was sufficient to capture the peaks. A smaller rotation step size Δϕ can also increase the number of domains that can potentially be detected, as we define each domain by its orientation. In other words, with smaller Δϕ, the sensitivity of detecting different domains or slight variation in the orientations is increased. Lastly, the instrument resolution associated with the incident beam should be much smaller than Δϕ, which was the case in this work, to ensure that the peaks were captured correctly at corresponding angles.

In this first demonstration of GID tomography for mapping crystalline domain orientations, the experiment did not require any modification to beamline setups in terms of both the hardware equipment and the software control. The data acquisition speed can be dramatically improved if the acquisition routine is modified to adopt a fly scan so as to remove the current speed bottleneck of long motor movement and settling time. The beam size and step size in *x* can also be optimized to reduce total data acquisition time by considering the domain sizes and the resolution limit imposed by Φ_crys_.

### 4.2. Comparison with existing characterization methods   

The characterization of thin films can require different geometries, including transmission/reflection and grazing incidence. The small probe in the transmission geometry offers high spatial resolution but the sample size is limited to the length scale of the probe. Scanning electron microscopy (SEM) and focused ion beam SEM provide nanoscopic resolution with the caveat of limitations on the choices of substrate and the scanning dimensions. Electron backscatter diffraction allows the study of crystal orientations but may not be suitable for organic materials, such as the thin films studied in this work, owing to the strong scattering from the Si substrates and the material’s susceptibility to beam damage. Polarized Raman spectroscopy shows promising resolution for studying the in-plane morphology of thin films (Bhardwaj *et al.*, 2019 ▸; Huang *et al.*, 2019 ▸). Raman signals are not directly related to crystal structures but are results of the correlation between molecules, which may restrict the choices of materials. Polarized optical microscopy provides in high resolution the morphology of thin films in both transmission and reflection geometries. Careful calibrations may need to be taken into account for accurate quantitative characterization, especially in the reflection geometry because of the additional propagation and reflection. Reflection high-energy electron diffraction and low-energy electron diffraction (LEED) are surface-sensitive methods that require vacuum environments, and samples often go through heat treatment to ensure a clean surface. This thermal treatment (353–373 K) can alter the structure for some materials or melt the sample entirely. For example, the BTBT films used here change phase and melt at around 343 K. For LEED, the material needs to be conductive or only a few nanometres thick on a conductive substrate, which severely limits the material that can be studied or the use of coatings that are necessary, for example, to help crystallization or alignment. Transmission X-ray scattering provides direct proof for in-plane orientations but, as a result of the distorted *q* space probed, each scattering pattern reveals only one or very few diffraction peaks. On the other hand, methods based on the grazing-incidence geometry sacrifice the spatial resolution and probe a large area of the sample with an elongated footprint to enhance the detected signal level. Most significantly, the GI geometry removes the substrate restriction for transmission measurements and the need for the material of interest to be isolated or have specific properties. Another advantage of GI methods is the ability to control the penetration and investigate the depth profile of films by varying the grazing-incidence angle.

GIWAXS is one of the most commonly used techniques to reveal structures in both in-plane and out-of-plane directions. GID tomography combines the advantage of the GI measurement geometry and the resolving power of tomographic methods to reconstruct the spatial position and shape of each domain as well as the crystalline orientation within each domain. Instead of accepting the sacrificed resolution due to the elongated X-ray footprint, GID tomography takes advantage of the projection information acquired by this footprint and utilizes it for tomography. The method provides a direct means to image material morphology as it builds on GIWAXS data sets, which provide direct information on crystal orientation.

### 4.3. Advanced material characterization   

In this work, each 1D projection in the domain sinogram was transformed by normalization with respect to its maximum, as shown by equation (3). This simple processing disregards the structure factor but still captures the spatial distribution of domains and offers successful reconstructions. It is possible to obtain a more accurate formation of the domain sinogram if the structure factor of the crystal can be considered to carefully adjust the intensity of each 1D projection (Breiby *et al.*, 2008 ▸). With a rigorous intensity calibration, reconstructed domains will not only offer quantitative orientation maps but also provide information on the crystallinity, which is not necessarily proportional to the domain size. Higher crystallinity would translate to higher intensity in a domain sinogram compared with that of other domains.

The BTBT films studied here had the same out-of-plane orientation and the different in-plane orientations were reconstructed. It is also possible to use GID tomography for reconstructing 3D morphology of thin films with varying orientations in **z** by utilizing other advantages of GIWAXS. For example, a depth profile of the film can be established by varying the grazing-incidence angle. In addition, GIWAXS allows for the identification of different orientations of thin films by indexing the corresponding scattering peaks and calculating the component ratio of *e.g.* in- and out-of-plane orientations (Ward *et al.*, 2014 ▸). GID tomography can be further developed on the basis of these features to enable 3D reconstruction of thin films.

Polymorphism is often expected in materials, for instance in the constrained area near the grain boundaries in organic transistor materials (Li *et al.*, 2016 ▸). GID tomography has the potential to account for polymorphs or mixed materials by having a multiple crystal rotation list. Realizing this potential requires high resolution in real space to resolve the domain boundaries, as well as high resolution in the *q* space to distinguish closely located peaks that originate from different polymorphs and formulate the correct crystal rotation lists. The detector position needs to be optimized to allow for sufficient *q*-space resolution to identify polymorphs and enough *q* range to cover enough peaks for adequate angular sampling for tomography. Alternatively, several detector positions can be scanned or multiple detectors can be used for simultaneous data acquisition.

## 5. Conclusion   

GID tomography presents a panoramic view of the structure and morphology of thin films – domain shapes and absolute orientations are revealed for centimetre-sized films in this work. We have shown the successful reconstruction of the in-plane structures for BTBT thin films on transparent and nontransparent substrates. Other characterization methods offer means to determine thin-film crystal orientations, each with different constraints on the material properties or sample preparation procedures. GID tomography is demonstrated as a nondestructive method to reveal the interrelation between the morphology and crystalline structure over multiple length scales without constraints on the substrate type or material thickness. Implemented at a bending-magnet beamline with standard X-ray scattering setup, GID tomography has minimal requirements on instrument resolution and coherence, while it takes advantage of the unparalleled photon flux and acquisition speed at synchrotron beamlines. The computational nature of the method means that it can easily expand the capabilities of existing X-ray scattering beamlines.

## Supplementary Material

An example of the raw GIWAXS data. DOI: 10.1107/S1600576721007184/vh5142sup1.avi
